# Seroprevalence of Asymptomatic* Leishmania donovani* among Laborers and Associated Risk Factors in Agricultural Camps of West Armachiho District, Northwest Ethiopia: A Cross-Sectional Study

**DOI:** 10.1155/2018/5751743

**Published:** 2018-11-28

**Authors:** Animen Ayehu, Yibeltal Aschale, Wossenseged Lemma, Animut Alebel, Ligabaw Worku, Ayalew Jejaw, Abebe Genetu Bayih

**Affiliations:** ^1^Department of Medical Parasitology, College of Medicine and Health Sciences, Bahir Dar University, Bahir Dar, Ethiopia; ^2^Department of Medical Parasitology, College of Medicine and Health Sciences, Debre Markos University, Debre Markos, Ethiopia; ^3^Department of Medical Parasitology, School of Biomedical and Laboratory Sciences, College of Medicine and Health Sciences, University of Gondar, Gondar, Ethiopia; ^4^Department of Advanced Clinical Pediatrics and Child Health Nursing, College of Medicine and Health Sciences, Debre Markos University, Debre Markos, Ethiopia

## Abstract

**Background:**

Visceral leishmaniasis (VL, also called kala-azar) is a public health problem in Ethiopia, especially in sesame and sorghum growing areas. Compared to other populations, labor migrants are the most exposed. Knowing the seroprevalence of* Leishmania donovani* and associated risk factors is essential to design appropriate control measures. The main aim of this study was to assess the seroprevalence of asymptomatic* L. donovani* among laborers and associated risk factors in agricultural camps of West Armachiho district, Northwest Ethiopia. Therefore, this study was conducted to know the seroprevalence and associated risk factors of* L. donovani* infection.

**Method:**

A cross-sectional study was conducted among 185 laborers from October to December 2017. A simple random sampling technique was used to select study participants from selected agricultural camps. After obtaining written informed consent, data were collected using a structured pretested Amharic version questionnaire using the interview technique. A single finger prick blood sample was collected from the study participants and the blood samples were subjected to the serological diagnostic method using the rk39 kit. The multivariable logistic regression model was used to identify risk factors associated with* L. donovani* infection.

**Result:**

Among 185 participants examined using rk39, 14 (7.6%) were seroreactive for* L. donovani*.* Leishmania donovani* infection had a statistically significant association with sleeping under* Balanites* trees (AOR: 4.36, 95%CI: 1.186-16.06), presence of domestic animals near sleeping place (AOR: 4.68, 95% CI: 1.25-17.56), and lack of knowledge about VL transmission (AOR: 3.79, 95% CI: 1.07-13.47).

**Conclusion:**

Seroprevalence of asymptomatic* L. donovani* among laborers in agricultural camps of West Armachiho was low. Prevention measures and health education about risk factors that expose to* L. donovani* infection for the laborers are essential to prevent the spread of the disease.

## 1. Introduction

Visceral leishmaniasis (VL, also known as kala-azar) is one of the major fatal vector-borne neglected tropical diseases caused by the protozoan parasites of the genus* Leishmania* (order: Kinetoplastida, family: Trypanosomatidae) [1]. Visceral leishmaniasis is endemic in more than 79 countries and causes an estimated annual incidence of 202,000-400,000 clinical cases and about 20,000-40,000 deaths per year worldwide [2]. Ethiopia is one of the six countries (Bangladesh, Brazil, Ethiopia, India, Nepal, and Sudan) in which more than 90% of global VL cases occur with the burden of 3,700-7,400 new cases per year [1, 2]. It is transmitted between humans and other mammalian hosts by the bite of infected female sand flies of the genus* Phlebotomus* or* Lutzomyia* (order: Diptera, family: Phlebotominae/Psychodidae) in the Old World and New World, respectively [3]. Visceral leishmaniasis is a disease of the reticuloendothelial system (liver, spleen, bone marrow, and lymph nodes) caused by an obligate intracellular* Leishmania donovani *complex parasite flashing its life cycle between the mammalian host (amastigote) and phlebotomine sand fly (promastigote) [1, 3]. It is the most severe form of leishmaniasis and if left untreated it causes death [4].

In Ethiopia,* Leishmania donovani* is the principal cause of VL [5], although* L. infantum* was identified in six splenic aspirate isolates during an outbreak in Libo Kemkem, Amhara region [6].* Phlebotomus orientalis *Parrot and* Phlebotomus martini *Parrot are the main vectors of visceral leishmaniasis.* Phlebotomus celiae *Minter is a probable secondary vector in narrow foci of Ethiopia and Kenya [7, 8].* Phlebotomus orientalis* is confined to specific habitats, with high abundance of* Acacia seyal *and* Balanites aegyptiaca *trees that are growing in crack-black cotton-clay soil [9, 10].

In northern Ethiopia, where the largest kala-azar focus (Metema-Humera lowlands) is found, the prevalence of VL is gradually rising posing an increasing public health concern. In this region, VL particularly affects migrant workers [11], in addition to residents involved in agricultural activities [12]. A total of 1,258 VL cases were treated from 2009 to 2011 in Kahsay Abera Hospitals in Humera [11]. The region has recently experienced epidemics in previously unaffected areas [1, 11] and long recognized VL endemic foci situated in Metema and Humera along the border with Sudan in the northwest [13]. Several outbreaks of visceral leishmaniasis have occurred between 2005 and 2008. A documented outbreak of VL with 2,500 cases and very high mortality rate occurred in Amhara region, particularly in Libo Kemkem [14, 15]. By 2007, around 2,450 primary cases and 120 deaths had been reported [15, 16].

In endemic areas,* L. donovani* infection does not mean clinical illness. Due to reasons not well understood, in Eastern Africa,* L. donovani* infection remains asymptomatic in certain subjects and causes lethal disease in others. The ratio of incident of asymptomatic to clinical cases in Ethiopia is 5.6:1 [17] compared to the range from 1:2.6 to 11:1 in Sudan [18] and 4:1 in Kenya [19].

In Ethiopia, economic impact of VL is due to high cost of treatment and time lost during hospitalization. Visceral leishmaniasis affects the rural poor community and usually outbreaks occur during harvesting seasons [14]. Seasonal migration between endemic and nonendemic regions, combined with biological, environmental, and socioeconomic risk factors, is responsible for the spread of the disease [14, 20]. Visceral leishmaniasis remains a public health problem particularly in migrant and nonmigrant laborers involved in agricultural activities in Metema-Humera including West Armachiho district. Due to growing of mechanized agricultural activities, which engage a large number of laborers, VL cases are increasing. Some of the morbidity and mortality that occurred during epidemic time of areas surrounding Metema-Humera lowlands could be related to lack of preparedness to prevent VL. This study was conducted to know the prevalence of* L. donovani *infection among laborers and associated risk factors in agricultural camps: which, in turn, is important to design and implement successful VL control and prevention programs. Risk factors like sociodemographic characters and behavioral and environmental factors were analyzed to show the risk factors for the infection of* L. donovani* in West Armachiho district agricultural camps.

## 2. Materials and Methods

### 2.1. Study Area and Study Period

The study was conducted in West Armachiho district agricultural camps, North Gondar Zone, from October to December 2017. West Armachiho is located 200 kms from Gondar town and 930 kms from Addis Ababa, northwestern part of Ethiopia. According to the 2015 national census the woreda has a total population of 31,730, of whom 17,400 are men and 14,330 women; 15,075 or 47.5% are urban inhabitants. The average temperature, humidity, and elevation of the woreda are 38°C, 78%, and 638 m above sea level, respectively. West Armachiho is one of Ethiopia's most fertile agricultural zones (over 30 agricultural camps) with a large scale of farming of cash crops such as sesame, maize, cotton, and sorghum. Migrants travel to West Armachiho district to work in the weeding and harvesting seasons, mainly from July to August and September to November, respectively. West Armachiho district is endemic for VL and labor migrants are the most affected.

### 2.2. Study Design

A cross-sectional study design was carried out to determine the seroprevalence of asymptomatic* Leishmania donovani* among laborers and to assess associated risk factors in agricultural camps of West Armachiho district.

### 2.3. Data Collection Procedure

#### 2.3.1. Demographic Data

Data were collected by a structured pretested Amharic version questionnaire using the interview technique. Data collection was carried out by the principal investigator and coinvestigator. Data collectors conducted a camp-to-camp survey. At each data collection spot, sufficient explanation about the aim of the research was given to the study participants before the interview.

#### 2.3.2. Sample Collection Procedure

A single finger prick blood specimen (8-12*μ*L) was collected from each study participant. Each participant's finger was cleaned using alcohol and cotton before pricking the finger. Then the participant's finger was pricked, blood sample was taken, and diagnosis of* L. donovani* was performed by using the rk39 kit (IT LEISH, France). Finally, the result of the study participant was recorded on a recording sheet.

#### 2.3.3. Quality Control

All materials and procedures were adequately controlled. The questionnaire which was originally developed in English was translated to the Amharic language and back to English to ensure its consistency. The pretest was done in a camp similar to the study camps for the relevance and clarity of the questionnaire for the respondents. Negative and Positive contol samples were used to check the functionality of the rk39 kit used for the study.

#### 2.3.4. Data Processing and Analysis

All data were registered in a laboratory logbook during the study period, entered into EpiData version 3.1, and transferred to SPSS version 20 for analysis. Multivariate logistic regression was used to identify risk factors. Variables that had* p* value ≤ 0.2 in the bivariable analysis were included in the multivariable logistic regression analysis.* p* value less than 0.05 was taken as statistically significant.

## 3. Results

### 3.1. Sociodemographic Characteristics of the Study Participants

In this study, 183 (98.9%) male and 2 (1.1%) female study participants were enrolled with mean (±standard deviation) and median (IQR) age of 26.02 (±8.65) and 24.0 (20-30), respectively (Table 1).

The majority of the study participants (60.5%) visited the area West Armachiho district for the second time and above and 39.5 % visited for the first time (Table 2).

### 3.2. Seroprevalence of* Leishmania donovani*

In this study, 183 male and 2 female study participants were enrolled and examined for seropositivity of* L. donovani* using the rk39 rapid diagnostic test. Out of the total, fourteen (male) study participants were reactive for rk39. The overall seroprevalence of asymptomatic* L. donovani* among laborers in West Armachiho agricultural camps was 7.6%. None of the two female study participants were reactive for the rk39 serological test. Seroprevalence of* L. donovani* between migrant laborers was 7.1% (10/140) and between resident laborers was 8.9% (4/45). Seroprevalence of* L. donovani* was 6.3% (6/95) within age group 15-24 years, 8.8% (6/68) within age group 25-34 years, and 9.1% (2/22) within age group ≥ 35 years. The seroprevalence of* L. donovani* within those who were unable to read and write, had primary school education, and had secondary school education was 12.3%, 4.3%, and 6.2%, respectively. Among bed net users 2 (5.1%) respondents were reactive and the rest (94.9%) were nonreactive for the rk39 rapid diagnostic method.

### 3.3. Risk Factors Analysis Associated with Seroprevalence of* Leishmania donovani*

#### 3.3.1. Sociodemographic Risk Factors

In this cross-sectional study, sociodemographic risk factors for seroprevalence of* L. donovani* were analyzed in a bivariable analysis. Sociodemographic risk factors like age groups 25-34 years (COR: 1.43, 95% CI: 0.44-4.65, and* p* value: 0.54) and ≥35 years (COR: 1.48, 95% CI: 0.27-7.89, and* p* value: 0.64), etc. were analyzed and none of them had a statistically significant association (*p* value > 0.05) with increased seroprevalence of* L. donovani* infection (Table 3).

#### 3.3.2. Behavioral Risk Factors

Behavioral risk factors were also analyzed in the bivariable logistic regression analysis. Length of stay in the area, sleeping on vs. above the ground, sharing sleeping place with three or more people, not using mosquito nets, family history of infection with VL, and lack of knowledge about signs and symptoms of VL were not statistically associated with the outcome and not fitted for the multivariable analysis. The bivariable analysis of behavioral risk factors that had a statistically significant association with increased seroprevalence of* L. donovani* includes sleeping in agricultural fields (COR: 4.44, 95% CI: 1.01-19.47, and* p* value: 0.04), sleeping under* Balanites* trees (COR: 3.24, 95% CI: 1.04-10.12, and* p* value: 0.04), etc. (Table 3).

#### 3.3.3. Household and Environmental Risk Factors

Environmental risk factors, presence of black-cracked cotton soil near sleeping place (COR: 3.17, 95% CI: 1.05-9.57, and* p* value: 0.04) and presence of domestic animals near sleeping area (COR: 3.05, 95% CI: 1.01-9.23, and* p* value: 0.04), had a statistically significant association with the increased seroprevalence of* L. donovani* in the bivariable analysis (Table 3).

Variables that were statistically associated with seroprevalence of* L. donovani* and had* p* value ≤ 0.2 in the bivariate analysis were analyzed by a multivariable analysis. Behavioral risk factors, sleeping under* Balanites* trees (AOR: 4.36, 95% CI: 1.18-16.06, and* p* value: 0.02) and lack of knowledge about transmission of VL (AOR: 3.79, 95%CI: 1.06-13.47, and* p* value: 0.03), and an environmental risk factor, presence of domestic animals near sleeping place (AOR: 4.68, 95% CI: 1.24-17.56, and* p* value: 0.02), were statistically significant risk factors for the increased seroprevalence of* L. donovani* in the multivariable analysis (Table 4).

## 4. Discussion

The risk of VL infection is high in immunologically naïve labor migrants who come from nonendemic highland areas compared with permanent residents in the towns of the lowland area [11]. Their involvement in agricultural fields where zoonotic VL cycle is maintained among unknown reservoir hosts could also make the incidence of VL high [21]. The overall seroprevalence of* L. donovani* among asymptomatic laborers in agricultural camps of West Armachiho district was consistent with the study conducted in Benishangul Gomez (7.3%) [22] and the study conducted in Libo Kemkem (6.5%) [23]. However, it was lower than the seroprevalence reported in Kafta-Humera (12.5%) [24]. The difference might have been because of the different serological diagnostic tests used in the studies. The serological test used in this study was rk39 (less sensitive than DAT) which might have decreased seroprevalence of* L. donovani* [25]. The other possible reason for the decreased seroprevalence of this study could be the different intervention strategies taken and awareness creation about the risk factors and transmission of VL by the Médecins Sans Frontières (MSF) Center. Smaller sample size used in this study might be the reason for the decreased seroprevalence of* L. donovani* infection.

In this study, the seroprevalence of* L. donovani* was much lower than in the study conducted in Bangladesh (29.2%) [26]. The difference might be due to the residence status of study participants. The study participants in Bangladesh were residents and had a great chance of being infected with* L. donovani* since they lived in the area throughout the year, while the study participants of the present study were migrants who come from nonendemic areas and stay in the study area for a short period of time in which transmission of* L. donovani* is low due to low density of* P. orientalis* because of rain. The study participants enrolled in Bangladesh had also history of contact with VL patients, but the study subjects enrolled in this study might not have had contact with VL patients, which might have decreased seroprevalence of* L. donovani*. The third option might be the fact that the study conducted in Bangladesh was a one-year study, but the present study was a short-period cross-sectional study, which might have decreased the seroprevalence of* L. donovani *infection.

The seroprevalence of* L. donovani* revealed by the present study was also lower than reported in Nepal (13.4%) [27]. The possible reason for the discrepancy of the two prevalence values might be due to the difference in endemicity of the areas and sampling techniques used. The sampling technique used in Nepal was the nonprobability sampling technique, while the sampling technique used in this study was the proportionate simple random sampling, which might have decreased the seroprevalence of* L. donovani* infection. The other option which contributes to the difference might be the status of the study participants. The study participants (8%) enrolled in Nepal had previous history of VL, while the study participants enrolled in this study had no previous history of VL, which might have decreased seroprevalence of* L. donovani.*

In this study different risk factors which contributed to the increased seroprevalence of* L*.* donovani* in the study area were assessed in the multivariate analysis using SPSS version 20. The study revealed that sleeping under* Balanites* trees was significantly associated with the increased seroprevalence of* L. donovani* infection. This study finding is supported by the retrospective case control study conducted in Northwest Tigray [12]. Laborers who slept under* Balanites *trees were at a higher risk of being infected with* L. donovani* than those who did not sleep under* Balanites* trees. This might be due to the fact that sand fly* P. orientalis* uses the habitat as a resting site and gets access to bite study participants while they sleep under it.

The presence of domestic animals near sleeping place was statistically associated with the increased seroprevalence of* L. donovani* in this study. This finding is supported by the studies conducted in Bangladesh, Libo Kemkem, and Tigray [12, 23, 26]. Study participants who slept near domestic animals had a higher chance of being infected with* L. donovani* than those who did not sleep near domestic animals. The reason that presence of domestic animals near sleeping place has increased the odds of* L. donovani* seroprevalence might be the zoonotic nature of the disease [21] and needs further studies on the behavior of sand flies and parasite to confirm the zoonotic nature of VL in the study area. This study noticed that sleeping place of migrants should be far from domestic animals.

This study also showed that lack of knowledge about transmission of VL was a statistically significant risk factor for the increased seroprevalence of* L. donovani* infection. Laborers who did not have knowledge about VL transmission were 3.79 times more likely to be infected with the etiological agent of VL. The increment of seroprevalence of* L. donovani* infection due to lack of knowledge about VL transmission might be due to the fact that laborers who know how and by what* L. donovani* is transmitted might use different mechanisms (buying bed net, using repellants, and covering their whole bodies by clothes while they slept) to prevent the bite of infected female* P. orientalis*, but laborers who had no knowledge about transmission of VL might not use similar mechanisms to prevent the bite of infected female* P. orientalis* sand flies, which in turn increased seroprevalence of* L. donovani* infection.

## 5. Conclusion

*Leishmania donovani* infection among asymptomatic laborers in agricultural camps of West Armachiho was lower than the prevalence revealed in Kafta-Humera. Risk factors statistically associated with increased seroprevalence of* L. donovani* were sleeping under* Balanites* trees, presence of domestic animals near sleeping place, and lack of knowledge about transmission of visceral leishmaniasis. Mass screening of asymptomatic laborers helps to decrease transmission of the disease, since carriers transmit the disease.

## 6. Limitations of the Study

The major limitation of this study was it being a cross-sectional study, and hence it cannot identify when the study subjects were infected. The other limitation of this study is that microscopic diagnosis for the participants was not performed due to need for expert personnel to take bone marrow aspirate and the diagnostic test used was nonspecific.

## Figures and Tables

**Table 1:** Sociodemographic characteristics of the study participants.

**Variables**	**Characteristics**	**Frequency**	**Percent (**%**)**
Sex			
	Male	183	98.9
	Female	2	1.1
Age			
	15-24	95	51.3
	25-34	68	36.8
	≥35	22	11.9
Educational status			
	Unable to read and write	56	30.3
	Primary	48	25.9
	Secondary	81	43.8
Occupation			
	Weeding and harvesting	170	91.9
	Driver	1	0.5
	Sheep and cattle keeping	12	6.5
	Food cooking	2	1.1
Residence			
	Resident laborers	45	24.3
	Migrant laborers	140	75.7

**Table 2:** Frequencies and percentages of possible risk factors for seroprevalence of asymptomatic *Leishmania donovani*.

Variables	Characteristics	Frequency	Percent
Length of stay in the area			
	Three months	82	43.4
	Six months	39	21.1
	One year	19	10.3
	Two years and above	45	24.3
Visiting frequency			
	First	73	39.5
	Second	33	17.8
	Third	35	18.9
	Fourth and above	44	23.8
Sleeping habit			
	Indoor	84	45.4
	In agricultural fields	22	11.9
	Subshelter	79	42.7
Sleeping condition			
	On the ground	79	42.7
	Above the ground	106	57.3
Shares sleeping place with three or more people			
	Yes	31	16.8
	No	154	83.2
Presence of crack soil near sleeping place			
	Yes	48	25.9
	No	137	74.1
Presence of domestic animals near sleeping place			
	Yes	60	32.4
	No	125	67.6
Sleeping under *Balanites *trees			
	Yes	70	37.8
	No	115	62.2
Uses bed net			
	Yes	39	21.1
	No	146	78.9
Travel history to other VL endemic areas			
	Yes	71	38.4
	No	114	61.6
Family history of infection with VL			
	Yes	44	23.8
	No	141	76.2
Knows signs/symptoms of VL			
	Yes	128	69.2
	No	57	30.8
Knows transmission of VL			
	Yes	127	68.6
	No	58	31.4

**Table 3:** Bivariable logistic regression of risk factors in relation to the seroprevalence of asymptomatic* Leishmania donovani*.

Seroprevalence of *L. donovani*			Statistics	

Variables	Pos (%)	Neg (%)	COR (95% CI)	*p* value

**A. Behavioral risk factors**				
**1.** Sleeping habit				
Indoor	4 (4.8)	80 (95.2)	1.00	
In agricultural fields	4 (18.2)	18 (81.8)	4.44 (1.01-19.47)	0.04*∗*
Subshelter	6 (7.6)	73 (92.4)	1.64 (0.44-6.05)	0.45
**2.** Sleeping under *Balanites* trees				
Yes	9 (12.9)	61 (87.1)	3.24 (1.04-10.12)	0.04*∗*
No	5 (4.3)	110 (95.7)		
**3. **Travel history to other VL endemic areas				
Yes	9 (12.7)	62 (87.3)	3.16 (1.01-9.86)	0.04*∗*
No	5 (4.4)	109 (95.6)		
**4. **Knows about transmission of VL				
No	8 (13.8)	50 (86.2)	3.22 (1.06-9.77)	0.03*∗*
Yes	6 (4.7)	121 (95.3)		
**B. Household and environmental risk factors**				
1. Presence of crack-black cotton soil near sleeping place				
Yes	7 (14.6)	41 (85.4)	3.17 (1.05-9.57)	0.04*∗*
No	7 (5.1)	130 (94.9)		
2. Presence of domestic animals near sleeping place				
Yes	8 (13.3)	52 (86.7)	3.05 (1.01-9.23)	0.04*∗*
No	6 (4.8)	119 (95.2)		

*∗*= statistically significant (*p* < 0.05).

**Table 4:** Multivariable logistic regression of associated risk factors for the seroprevalence of asymptomatic *Leishmania donovani*.

**Seroprevalence of *L. donovani***			**Statistics**	

**Variables**	**Pos (**%**)**	**Neg (**%**)**	**AOR (95**%** CI)**	***p* value**

**A. Behavioral risk factors**				
1. Sleeping under *Balanites *trees				
Yes	9 (12.9)	61 (87.1)	4.36 (1.18-16.06)	0.02
No	5 (4.3)	110 (95.7)		
2. Knows transmission of VL				
No	8 (13.8)	50 (86.2)	3.79 (1.06-13.47)	0.03
Yes	6 (4.7)	121 (95.3)		
B. Household risk factor				
1. Presence of domestic animals near sleeping place				
Yes	8 (13.3)	52 (86.7)	4.68 (1.24-17.56)	0.02
No	6 (4.8)	119 (95.2)		

## Data Availability

The data used to support the findings of this study are available from the corresponding author upon request.
